# *Abriendo Puertas*: Baseline Findings from an Integrated Intervention to Promote Prevention, Treatment and Care among FSW Living with HIV in the Dominican Republic

**DOI:** 10.1371/journal.pone.0088157

**Published:** 2014-02-14

**Authors:** Yeycy Donastorg, Clare Barrington, Martha Perez, Deanna Kerrigan

**Affiliations:** 1 HIV Vaccine Research Unit, Instituto Dermatalógico y Cirugia de Piel Dr. Humberto Bogart Diaz, Santo Domingo, Rep. Dom; 2 Department of Health Behavior, Gillings School of Global Public Health, University of North Carolina at Chapel Hill, Chapel Hill, North Carolina, United States of America; 3 Department of Health, Behavior and Society, The Johns Hopkins Bloomberg School of Public Health, Baltimore, Maryland, United States of America; Alberta Provincial Laboratory for Public Health/University of Alberta, Canada

## Abstract

Female sex workers (FSW) are often the focus of primary HIV prevention efforts. However, little attention has been paid to the prevention, treatment, and care needs of FSW living with HIV. Based on formative research, we developed an integrated model to promote prevention and care for FSW living with HIV in Santo Domingo, Dominican Republic, including (1) individual counseling and education; (2) peer navigation; (3) clinical provider training; and (4) community mobilization. We enrolled 268 FSW living with HIV into the intervention and conducted socio-behavioral surveys, sexually transmitted infection (STI) testing, and viral load (VL) assessments. We used multivariate logistic regression to identify behavioral and socio-demographic factors associated with detectable VL (>50 copies/mL) and STI prevalence. Over half of all participants (51.9%) had a detectable VL, even though most received HIV-related care in the last 6 months (85.1%) and were currently on anti-retroviral treatment (ART) (72.4%). Factors positively associated with a detectable VL included being 18–35 years of age (Adjusted Odds Ratio [AOR] 2.46, 95% CI 1.31–4.60), having ever used drugs (AOR 2.34, 95% CI 1.14–4.79), and having ever interrupted ART (AOR 3.09, 95% CI 1.44–6.59). Factors protective against having a detectable VL included being single (AOR 0.45, 95% 0.20–0.98) and being currently on ART (AOR 0.17, 95% CI 0.07–0.41). Nearly one-quarter (23.1%) had an STI, which was associated with being single (AOR 3.21, 95% CI 1.27–8.11) and using drugs in the last 6 months (AOR 3.54, 95% CI 1.32–9.45). Being on ART was protective against STI (AOR 0.51, 95% CI 0.26–1.00). Baseline findings indicate significant barriers to VL suppression and STI prevention among FSW living with HIV and highlight gaps in the continuum of HIV care and treatment. These findings have important implications for both the individual health of FSW and population-level HIV transmission dynamics.

## Introduction

The heightened burden of HIV among female sex workers (FSW) has been well documented across geographic settings and epidemic dynamics [1], [2]. To date, most HIV-related efforts with FSW have focused on primary prevention and surveillance [3], [4]. Minimal attention has been paid to the needs of FSW living with HIV, their access to quality care services, and their treatment outcomes [5]. Improving access to quality HIV care and treatment services for FSW is first and foremost a human rights issue as all people living with HIV have the right to care and treatment [6]. More recently, the population-level prevention benefits of antiretroviral therapy (ART) in suppressing viral load (VL) and minimizing HIV transmission in discordant couples, or “treatment as prevention” (TasP), have been firmly established [7], [8]. There may be additional public health prevention benefits to ensuring access to quality HIV care and treatment services for key populations such as FSW who have large sexual networks [9]–[13].

FSW living with HIV face multiple layers of stigma and discrimination including but not limited to their HIV status, occupation, socio-economic position, and inequitable gender and sexuality roles and norms [14], [15]. In turn, these populations may need additional support systems to address the psychosocial and structural barriers associated with linkages to and retention in quality HIV care services and adherence to ART, in addition to policy-level and social change efforts [16], [17]. The limited research on this topic to date has documented stigma and discrimination as barriers to care and treatment among FSW in a variety of settings [5], [15], [18]–[20]. Among people living with HIV, having a history of sex work has been found to be significantly related to experiencing HIV-related stigma in Brazil [21]. Additionally, Diabaté et al. [22] found that FSW in Benin had lower adherence and a slower response to treatment compared to individuals living with HIV who were not involved in sex work, suggesting that FSW may experience unique challenges and barriers to achieving optimal HIV treatment outcomes. Among a cohort of FSW living with HIV in Kenya, however, McClelland et al. [23] reported no increases in sexual risk behavior following initiation of ART, which was attributed to the context of ongoing risk reduction education and condom availability.

The HIV epidemic in the Dominican Republic (DR) is concentrated, with a disproportionate burden of disease among FSW, men who have sex with men, and drug users [24], [25]. Despite its small overall population size, the DR has a large female sex industry, with tens of thousands of women working in a variety of sex work settings and modalities within the country [26]. The most recent estimates of HIV prevalence among FSW indicate that prevalence varies significantly across cities and regions, ranging from 3.3% in Santo Domingo, where ongoing HIV prevention efforts have existed since the late 1980s, to 8.4% in the Southwestern city of Barahona, where there have been minimal HIV prevention interventions to date [25], [27].

Community-based, environmental-structural HIV prevention efforts with FSW in the DR have achieved significant improvements in condom use and declines in sexually transmitted infections (STI) among sex workers [28]. However, until recently almost no research or programs have addressed the experiences of FSW living with HIV in the DR. We conducted formative, qualitative research with FSW living with HIV in Santo Domingo, the capital city, and documented substantial barriers related to sustaining engagement in HIV treatment and care due to layered stigma and discrimination, delayed diagnosis and lack of post-test counseling, involuntary disclosure of HIV status by clinic staff, and poor quality of services in clinics [29]. Women also described considerable HIV care and treatment-related expenses, including the high costs of transportation to get to appointments and prescribed medicines and treatments beyond ART. Additionally, while participants were very aware of the need to prevent HIV transmission to sexual partners, many reported that economic hardship made it difficult to practice preventive behaviors, such as when clients offered more money in exchange for sex without condoms, as has been described by McClelland et al. [20] based on results of a study with FSW living with HIV in Kenya. Also related to the economic impact of living with HIV, we found that several participants had only become FSW after their diagnosis, when they were unable to find other work due to discriminatory employment-related HIV screening and increased economic needs.

In response to these findings and the larger gap in the literature regarding the needs and experiences of FSW living with HIV, a collaborative partnership of academic, clinical care, non-governmental organizations (NGO), and community groups established the *Abriendo Puertas*, or Opening Doors, intervention research project to improve HIV/STI outcomes and overall well-being among FSW living with HIV in the DR.

## Methods

### Study setting and design

*Abriendo Puertas* is an ongoing longitudinal intervention research project that aims to assess the initial effects of an integrated intervention to promote HIV care and preventive behaviors on HIV outcomes (e.g., VL, STI) and behaviors (e.g., consistent condom use, engagement in care). We are using a mixed methods approach to document the level and nature of exposure to and participants' experiences with different components of the intervention including structured socio-behavioral surveys, qualitative in-depth interviews, and focus groups. In this paper, we will present findings from the baseline socio-behavioral quantitative survey.

### Ethics section

The study is being conducted at the HIV Vaccine Research Unit (HVRU) at the Instituto Dermatalógico y Cirugia de Piel Dr. Humberto Bogart Diaz (IDCP) in Santo Domingo. The intervention is implemented in partnership with the Movimiento de Mujeres Unidas (MODEMU), a sex worker rights organization in Santo Domingo, as well as the Centro de Orientacion e Investigacion Integral (COIN), an NGO that pioneered HIV prevention efforts with FSW in the DR. In order to protect participant confidentiality, participants were required to provide only oral consent. Trained interviewers signed consent forms in lieu of participants, indicating that they had reviewed the form with participant, the participant had no further questions, and the participant agreed to enroll in the study. All study protocols and consent procedures were approved by the Institutional Review Boards of the Johns Hopkins Bloomberg School of Public Health and the IDCP.

### Sample and recruitment

**Figure 1:**
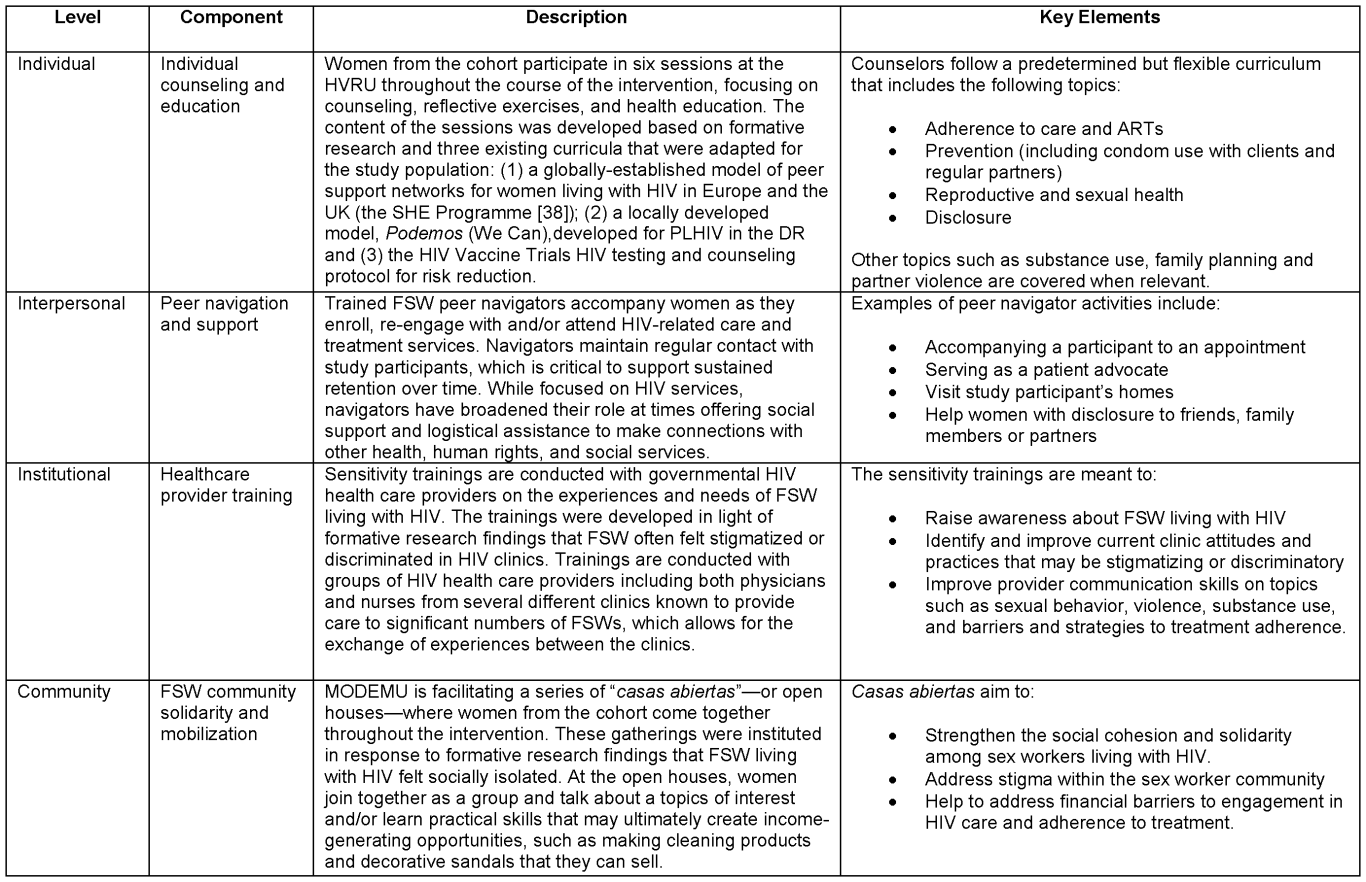
Intervention components of *Abriendo Puertas*.

FSW in this study are defined as women who report having exchanged sex for money in the last month. All participants were at least 18 years at the time of consent, spoke Spanish, and were HIV-positive. Participants were recruited through a non-random, hybrid sampling approach led by peer navigators, who were all current/former sex workers with experience doing HIV outreach, prevention, and support for people living with HIV (see Figure 1 for description of peer navigator role). Peer navigators approached women they already knew who were living with HIV through their ongoing community-based work with sex workers. In the HIV clinic context, peer navigators did short presentations about the study and provided their contact information for women who were interested to follow up. They also received referrals from clinic-based peer educators. Subsequent referrals were made by women who were participating in the study. Only women who self-reported during the recruitment process that they were living with HIV were invited to the study site; HIV status was confirmed before enrollment through a single rapid test (Retrocheck). From November 2012 to February 2013, we enrolled 268 FSW participants who are currently receiving the *Abriendo Puertas* intervention over a 10-month period. A total of 318 women were approached or inquired about participation in the study. Of these, 26 never came to the study site for a screening visit and 24 were determined to be ineligible, resulting in a baseline sample size of 268. The most common reason for ineligibility at screening was not being an active sex worker (71.0%).

### Data collection procedures

To assess the feasibility and initial effects of *Abriendo Puertas*, we conducted an interviewer-administered standardized socio-behavioral survey at baseline, which will be repeated at 10-month follow-up. All data collection occurs in Spanish in private offices of the HVRU by trained female Dominican field staff. Vaginal swabs for STI testing and whole blood samples for HIV VL testing were obtained during a clinical exam by a physician at baseline and will be repeated at 10-month follow-up. Vaginal swab specimens were processed at a CLIA-certified laboratory at the Johns Hopkins School of Medicine. Analyses were conducted using the Aptima Combo2 assay for gonorrhea and chlamydia and a separate trichomoniasis assay, all nucleic acid amplification testing (NAAT) methods. All participants who tested positive for STIs received treatment free of charge based on national standards of care. HIV VL was assessed at the Dominican National Reference Laboratory in Santo Domingo using polymerase chain reaction (PCR) testing.

### Measures

Biologic outcomes include having a detectable HIV RNA VL (defined at ≥50 copies/mL) and any prevalent STI (including gonorrhea, trichomoniasis, and chlamydia).

The socio-behavioral survey instrument includes questions on individual, relational, social-contextual, and structural factors and behaviors related to HIV prevention, care, and treatment outcomes. We drew on our previous research with FSW in the DR to inform the survey items and adapted measures from other settings as needed (described below). Independent variables included in the current analysis include socio-demographics (e.g., age, civil status, residence), history and type of sex work, number of new and regular clients in the last week and month, number of steady intimate partners in the last month, reported consistent condom use (defined as always using condoms in all sex acts with a defined partner category), drug and alcohol use, current or former use of ART, engagement in care (defined as attendance at any HIV care or treatment service in the last 6 months), any reported interruption in use of ART, and AIDS Clinical Trials Group (ACTG) measures related to adherence to ART [30].

We measured both internalized and experienced HIV stigma and discrimination using adapted measures from several reliable aggregate measures including those developed by Berger et al. [31], Zelaya et al. [32], [33] and Baral et al. [34]. We also employed Earnshaw's HIV Stigma Framework for the purposes of measurement, which defines internalized (or felt) stigma as the application of negative attitudes towards HIV to oneself and experienced (or enacted) stigma as the experience of being discriminated, stereotyped, or prejudiced against for being infected with HIV [35]. Aggregate measures for both domains demonstrated strong unidimensionality and internal reliability. The internalized stigma measure included 8 items assessed on a 4 point Likert-scale (score range 0 to 32, with 0 representing no stigma and 32 high internalized stigma) with a Cronbach's alpha of 0.87. Examples of internalized stigma items included feelings about living with HIV including whether they felt like people treated them differently or they felt like a bad or unworthy person as a result of their status. For the experienced stigma score, there were 10 items with yes or no answers to each (score range 0 to 10 with 0 representing no experienced stigma and 10 high experienced stigma) with a Cronbach's alpha of 0.78. Examples of experienced stigma included reported loss of job, denial of health and other social services, and being verbally harassed or physically abused as a result of living with HIV.

### Data analysis

Descriptive statistics including frequency distributions, medians, and ranges were calculated to characterize the study sample at baseline. Bivariate and multivariate logistic regression was utilized to assess unadjusted and adjusted associations between independent variables (e.g., sex work history, number of sex partners, consistent condom use, adherence to treatment, alcohol and drug use) and the two outcomes (VL, STI), controlling for socio-demographic characteristics. Independent variables with significant bivariate associations at the p<0.10 level were included in the multivariate models using a forward stepwise approach. All analyses controlled for socio-demographic characteristics (e.g., age, education, civil status, number of children, and city of residence). We present two multivariate logistic regression models developed to identify independent variables associated with VL, one among the whole sample of women and one among only women on ART, and one model to identify independent variables associated with STI among all study participants. All statistical analyses were conducted in SPSS version 21.

### Intervention description

*Abriendo Puertas* is an integrated HIV care and prevention intervention with four components. We designed the intervention based on findings from our formative research with FSW living with HIV in which we found individual, social-contextual, and structural barriers to engagement and retention in care and adherence to treatment. Our model is informed by the concept of layered or intersecting stigmas [36], [37]. In their framework on HIV stigma, Aggleton and Parker [36] position stigma as a social mechanism for reinforcing existing differences that, “feeds upon, strengthens and reproduces existing inequalities of race, gender, and sexuality” (p. 13). In our formative research, we found that women described experiencing multiple, overlapping forms of stigma and discrimination related to their identities as sex workers, being individuals living with HIV, being women, and their socio-economic status.

The intervention's four key components are simultaneously implemented during the 10-month follow-up period. These include: (1) individual counseling and education, based on formative research results and existing intervention models [38]; (2) peer HIV service navigation and support; (3) clinical health care provider sensitivity training; and (4) community solidarity and mobilization. These components provide a multi-level response to the social and structural context surrounding the stigma, discrimination, and fear experienced by FSW living with HIV. Each element is described in more conceptual and operational detail in Figure 1.

## Results

### Socio-demographic, behavioral, and biological characteristics of the sample

As reflected in **Table 1**, the median age of participants was 35.5 years (range 18–61), with the large majority (81%) being in some form of steady intimate partnership. Formal education was low and over one-third (35.4%) of participants had no secondary education. Almost all participants (93.7%) had at least one child with a median of 3 children (range 1–8). While most lived in Santo Domingo, 21.6% lived in other cities and rural areas of the country but came to Santo Domingo for their HIV care.

**Table 1:** Socio-demographic characteristics of Female Sex Workers Living with HIV at Baseline of the *Abriendo Puertas* Intervention in Santo Domingo (n = 268).

Variables	Percentage (frequency) in each category or median (range;IQR*)
Age in years	35.5 (18–61;12)
Civil status	
Single, no steady partner	19.0% (51)
Lives with a spouse/steady partner	38.4% (103)
Non-cohabitating steady partner	42.5% (114)
Education in years	7.00 (0–16;8)
0-8^th^ grade	64.6% (173)
9^th^ grade to university graduate	35.4%
Has any children	93.7% (251)
Number of children	3.0 (1–8;4)
Current residence	
Santo Domingo	78.4% (210)
Another city/town/rural area	21.6% (58)
Type of sex work venue	
Works in the street	56.6% (152)
Works in a sex establishment or independently	43.3% (116)
Years in sex work	15.00 (0–45;13)
Average price per date (in US dollars)	20 (5–100;12.50)
Alcohol use in last 30 days	
At least once a week	35.4% (125)
Less than weekly	64.6% (173)
Alcohol use before sex	
Sometimes/always	46.8% (125)
Never	53.2% (142)
Drug use ever	24.3% (65)
Drug use in last 6 months	8.2% (22)
Score for internalized HIV stigma	18.0 (8–32;17)
Score for experienced HIV stigma	1.0 (0–10;3)

*IQR = Interquartile Range

Participants worked in a range of sex work settings including the street, establishments, and independently via cell phone. Most had been in sex work for many years (median 15, range 0–45). Of interest, 18.8% of participants first became involved in sex work after their HIV diagnosis (data not shown), echoing findings from our formative work. While there was a wide range in the price that the women charged per date (5–100 $US dollars), the median charge per date was 800 pesos (or $US 20 dollars). Alcohol use was common among the sample and almost half (46.8%) of participants reporting using alcohol sometimes or always before having sex. Nearly one quarter of women (24.3%) reported ever having used drugs (marijuana, cocaine, crack, heroin); 8.2% reported drug use in the last 6 months. The median score for internalized stigma was 18 (range 8–32). For experienced stigma, the median score was 1 (range 0–10). Yet, 61.8% of participants reported one or more forms of experienced stigma or discrimination at some point (data not shown).

**Table 2** displays HIV-related behavioral and biological characteristics of our sample. The median number of reported sexual partners in the last month was 12 (range 1–51), with most women having at least one intimate partner and several regular clients, in addition to new clients. Condom use levels varied per these partner types, with consistent use being over 90 percent for both new and regular clients and 68.9% with steady intimate partners. Nearly one-quarter of the cohort (23.1%) had one or more STI at baseline; trichomoniasis was the most commonly diagnosed STI (20.6%).

**Table 2:** Behavioral and Biological Characteristics of Female Sex Workers Living With HIV at Baseline of the *Abriendo Puertas* Intervention in Santo Domingo, Dominican Republic (n = 268).

Variables	
*Sexual risk behaviors and outcomes*	Median (range;IQR*)
New clients in last 30 days	3.0 (0–49;5)
Regular clients in last 30 days	7.0 (0–46;7)
Steady partners in last 30 days	1.0 (0–9;0)
All sexual partners in last 30 days	12 (1–51;11.75)
	Percentage (N) per category
Consistent condom use with new clients in last 30 days	94.4 (201)
Consistent condom use with regular clients in last 30 days	93.1 (230)
Consistent condom use with steady partners in last 30 days	68.9 (153)
Consistent condom use with all partners in last 30 days	72.0 (190)
Presence of STIs	23.1 (57)
Chlamydia	3.2 (8)
Gonorrhea	0.8 (2)
Trichomoniasis	20.6 (51)
***HIV care and treatment behaviors and outcomes***	
Time since HIV diagnosis (in years)	5.0 (0–31)
Received HIV care in the last 6 months	85.1 (228)
Missed appointment in last 6 months (n = 228)	36.1% (82)
Ever taken ART	78.4 (210)
Currently taking ART	72.4 (194)
Ever interrupted ART	36.4 (76)
Adherence to ART last 4 days	
Missed at least 1 day	26.2 (55)
Never missed	73.8 (155)
Detectable viral load (≥50 copies/mL)	51.9% (138)
Exposure to intervention components in last 6 months	
Received individual counseling	29.5 (79)
Participated in social support group	35.4 (95)
Participated in activities related to rights of PLHIV	24.3 (65)

*IQR = Interquartile Range

The median reported length of time since HIV diagnosis was 5 years (range <1–31). While the majority had been engaged in care over the last 6 months, importantly, almost 15% had no contact with HIV-related care. Additionally, more than one-third (36.1%) of the sample that was engaged in care had missed a care appointment in the last 6 months. Approximately 72% were on ART at baseline. While adherence to ART in the last 4 days was relatively high (73.8%), 36.4% had interrupted ART at some point in the past. Additionally, 51.9% had a detectable VL at baseline.

Exposure to intervention components relevant to the *Abriendo Puertas* model was generally low at baseline, with 29.5% reporting having received any individual counseling, 35.4% participating in a social support group, and 24.3% participating in group-level activities related to the rights of PLHIV in the last 6 months.

### Associations with detectable viral load

Two models were developed to examine factors associated with detectable VL among study participants (**Table 3**). The first model included all participants whereas the second included just women who had ever been on ART. In bivariate analysis among all participants in the sample, being 18–35 (Unadjusted Odds Ratio [UOR] 3.71, 95% CI 2.23–6.16), having ever used drugs (UOR 3.48, 95% CI 1.88–6.47) and being diagnosed with HIV in the last 5 years (UOR 1.83, 95% CI 1.13–2.99) were positively associated with having a detectable VL. Being single compared to living with a steady partner (UOR 0.41, 95% CI 0.20–0.82) or compared to having a non-cohabitating steady partner (UOR .40, 95% CI 0.20–0.80), years in sex work (UOR 0.62, 95% CI 0.38–1.00), currently taking ART (UOR 0.15, 95% CI 0.08–0.29), and engagement in care (UOR 0.32, 95% CI 0.15–0.68) were all negatively associated (meaning protective against) having a detectable VL. Drinking alcohol at least once in the last week was significant at the p≤.10 level (UOR 1.56, 95% CI 0.94–2.59) and was included in the multivariate model. In multivariate analysis, younger age (18–35 years) (Adjusted Odds Ratio [AOR] 2.45, 95% CI 1.31–4.60), being single compared to having a non-cohabitating partner (AOR 0.45, 95% CI 0.20–0.98), having ever used drugs (AOR 2.34, 95% CI 1.14–4.79), and currently taking ART (AOR 0.17, 95% CI 0.07–0.41) remained significantly associated with having a detectable VL. While engagement in care did not remain significant in the multivariate model, it was significantly associated with currently being on ART (data not shown), and in turn, on the critical behavioral pathway to VL suppression.

**Table 3:** Unadjusted and Adjusted Odds of Having a Detectable Viral Load among Female Sex Workers Living with HIV at Baseline of the *Abriendo Puertas* Intervention in Santo Domingo, Dominican Republic.

	Among all women (n = 266)				Among women who have ever taken ART (n = 207)			
	UOR	95% CI	AOR	95% CI	UOR	95% CI	AOR	95% CI
***Socio-demographics***								
18–35 years old	3.71***	2.23–6.16	2.46**	1.31–4.60	3.53***	1.96–6.27	2.60**	1.29–5.24
Marital status								
-Single vs. lives with a steady partner	0.41**	0.20–0.82	0.50	0.25–1.47	**0.43** *	0.19–0.97	0.50	0.20–1.30
-Single vs. has a non-cohabitating single partner	0.40**	0.20–0.80	**0.45** *	0.20–0.98	**0.38** *	0.17–0.86	0.45*	0.18–1.09
0-8^th^ grade education	1.28	0.78–2.12	1.33	0.71–2.48	1.21	0.68–2.15	1.42	0.69–2.91
Has ≤2 children	1.38	0.85–2.23	0.95	0.53–1.72	1.41	0.81–2.46	0.99	0.51–1.93
Lives in Santo Domingo	1.26	0.70–2.26	1.20	0.59–2.42	1.48	0.76–2.90	1.58	0.71–3.51
***Sex work factors***								
Works in the street	1.11	0.68–1.80	—	—	1.23	0.71–2.14	—	—
Charges <$20 per date	1.14	0.70–1.84	—	—	1.36	0.78–2.35	—	—
Has been in sex work for ≥15 years	**0.62** *	0.38–1.00	0.83	0.43–1.59	0.58	0.34–1.01	0.75	0.36–1.57
***Behaviors***								
Drank alcohol at least once a week in the last 30 days	1.56	0.94–2.59	1.29	0.71–2.32	1.35	0.75–2.42	—	—
Ever used drugs	3.48***	1.88–6.47	**2.34** *	1.14–4.79	4.03***	1.99–8.15	**2.82** *	1.21–6.58
Diagnosed with HIV in the last 5 years	**1.83** *	1.13–2.99	1.20	0.67–2.15	**1.77** *	1.02–3.08	1.46	0.75–2.85
Had any HIV care in the last 6 months	0.32**	0.15–0.68	1.42	0.46–4.33	0.42	0.12–1.49	—	—
Currently taking ART	0.15***	0.08–0.29	0.17***	0.07–0.41	0.16**	0.04–0.57	0.48	0.11–2.19
No missed ART in the last 4 days	—	—	—	—	**0.49** *	0.27–0.92	1.37	0.59–3.21
Ever interrupted ART	—	—	—	—	3.55***	1.96–6.41	3.09**	1.44–6.59

*p≤0.05 **p≤0.01 ***p≤0.001

Among study participants who had ever been on ART, being 18–35 (UOR 3.53, 95% CI 1.96–6.27), having ever used drugs (UOR, 4.03, 95% CI 1.99–8.15), time since diagnosis (UOR 1.77, 95% CI 1.02–3.08), and interrupting ART (UOR 3.55, 95% CI 1.96–6.41) were all positively associated with having a detectable VL in bivariate analysis. In contrast, being single compared to living with a steady partner (UOR 0.43, 95% CI .19–0.97) or compared to having a non-cohabitating partner (UOR 0.38, 95% CI 0.17–0.86), and adherence to ART (UOR 0.49, 95% CI .27–.92) were protective. Years in sex work was significant at the p≤.10 level and was included in the multivariate model. In the multivariate analysis, we again saw the importance of age and drug use. Younger participants had a 2.60 greater odds of having a detectable VL (95% CI 1.29–5.24) compared to older participants, and women who had ever used drugs had a 2.82 greater odds of having a detectable VL (95% CI 1.21–6.58) than women who did not report any drug use. We also found that those who had interrupted ART at any point had a 3.09 greater odds of having a detectable VL than those who had not interrupted treatment (95% CI 1.44–6.59).

### Associations with having an STI

Several variables were positively associated with having an STI at baseline in bivariate analyses (**Table 4**), including having consumed alcohol at least once a week in the past month (UOR 1.94, 95% CI 1.08–3.49) and having used drugs in the past 6 months (UOR 4.27, 95% CI 1.73–10.62). Consistent condom use with all partner types in the past month (UOR 0.57, 95% CI 0.30–1.07) and currently taking ART (UOR 0.51, 95% CI 0.26–0.99) were protective. Of these variables, drug use and currently taking ART remained significant in multivariate analyses. Participants who reported drug use in the last 6 months had 3.54 greater odds of having an STI as compared to those who had not used (95% CI 1.32–9.45). Being on ART had a protective effect on STIs, such that those on ART had a 0.51 lower odds of having an STI (95% CI. 0.26–1.00) than those who were not on treatment. While not significant in bivariate analysis, civil status became significant in the multivariate model: Women who reported being single had a 3.21 times greater odds of having an STI at baseline than those who lived with a steady partner (95% CI 1.27–8.11).

**Table 4:** Unadjusted and Adjusted Odds of Having an STI among Female Sex Workers Living with HIV at Baseline of the *Abriendo Puertas* Intervention in Santo Domingo, Dominican Republic.

	Female Sex Workers Living with HIV (n = 257)			
	UOR	95% CI	AOR	95% CI
*Socio-demographics*				
18–35 years old	1.58	0.88–2.83	1.39	0.71–2.72
Marital status				
- Single vs. lives with a steady partner	1.83	0.81–4.09	3.21**	1.27–8.11
- Single vs. has a non-cohabitating steady partner	1.06	0.50–2.23	1.34	0.59–3.04
0-8^th^ grade education	1.49	0.79–2.79	1.51	0.75–3.06
Has ≤2 children	1.24	0.70–2.12	1.20	0.63–2.29
***Sex work factors***				
Works in the street	1.12	0.63–2.01	—	—
Charges <$20 per date	1.23	0.69–2.19	—	—
Has been in sex work for ≥15 years	0.85	0.48–1.51	—	—
***Behaviors***				
Drank alcohol at least once a week in the last 30 days	1.94*	1.08–3.49	1.48	0.78–2.82
Used drugs in the last 6 months	4.27**	1.73–10.62	3.54**	1.32–9.45
≥13 total sex partners in last month	1.48	0.83–2.65	—	—
Used condoms consistently with all partners in the last month	0.57	0.30–1.07	0.59	0.29–1.18
Currently on ART	0.51*	0.26–0.99	0.51*	0.26–1.00

*p≤0.05 **p≤0.01.

## Discussion

Baseline findings from the *Abriendo Puertas* study offer important insights into the prevention, treatment, and care needs of FSW living with HIV in the Dominican Republic, a population that has previously been understudied and underserved. Most strikingly, less than half of the women in our sample were virally suppressed, even though over 70% were on ART. Being on treatment and interruptions in treatment were both significantly associated with VL, highlighting the need to improve the quality and effectiveness of treatment among this population. While the majority of women were engaged in care in the last 6 months, we also found high levels of missed appointments, which could contribute to the lack of viral suppression. Diabate et al. [22] found that FSW living with HIV in Benin were slower to respond to ART than individuals not involved in sex work. Taken together, these findings suggest the need to improve understanding of and develop responses to barriers at the psychosocial, clinical, economic, and structural levels that shape the continuity, quality, and effectiveness of HIV treatment for FSW.

Interestingly, in both models we found that younger age (18 to 35 year) was significantly associated with having a detectable VL. These findings merit further investigation, but suggest some form of social vulnerability among younger women. Younger, recently diagnosed women may have more difficulty navigating the care and treatment service system. A detectable VL may also be a signal of lower adherence among younger women, as has been described in other settings [39]–[41]. In contrast, single women were significantly less likely to have a detectable VL than women with a non-cohabitating steady partner, suggesting that relationship dynamics may influence women's ability to adhere to treatment, which should be explored in future research.

Reported consistent condom use was high with new and regular clients. Consistent condom use with steady intimate partners was lower, though still the prevalent behavior. This phenomenon of lower rates of condom use with steady intimate partners has been documented previously [42]. Of interest is that the level of consistent condom use reported with steady intimate partners among this sample is much higher than past studies conducted among FSW in the DR and other settings [43], [44], suggesting that FSW living with HIV may be more successfully negotiating condom use within the confines of their ongoing intimate relationships than their negative counterparts. This is a similar trend to what has been observed in other general population studies indicating that people living with HIV have greater condom use than their HIV-negative counterparts [45].

Factors significantly associated with prevalent STI in multivariate analysis included three important variables: civil status, drug use, and being on ART. The finding that single women were at higher risk of STI than women who lived with a steady partner is of interest because relationship dynamics with steady partners could lead to less condom use and greater vulnerability to infection and ongoing transmission. While condom use dynamics with steady partners are still of relevance given the lower use reported in our cohort, attention must be paid to single women who may change partners more frequently, have more partners overall, and/or engage in concurrent partnerships, both of a commercial and non-commercial nature. It is also worth noting that women in our sample reported fairly high numbers of partners, potentially reflecting their extreme economic needs. Given the economic vulnerability of this population, it is important to continue identifying innovative ways to support women in negotiating consistent condom use with all partners as well as identifying supplemental forms of income due to the many direct and indirect costs associated with HIV care and treatment [15], [46], [47].

While overall less than a quarter of the sample reported ever using drugs and only 8% reported current drug use, those who did use drugs were at greater risk for STI, indicating the importance of integrating attention to drug use in the *Abriendo Puertas* intervention and providing linkages to drug-related services. Drug use among FSW is a highly sensitive and understudied topic in the DR. Our findings indicate the need for research on drugs, both as a risk factor for HIV infection as well as a potential determinant of ongoing transmission. Lastly, being on ART was significantly associated with not having an STI at baseline. This finding may be due to greater engagement with the HIV care and services system where they may be exposed to ongoing prevention messages and/or more opportunities for STI testing and treatment. It also suggests that being on ART is not linked to “treatment optimism”—the devaluing of the importance of safer sexual behaviors due to ART—among this population in this setting [48], [49].

FSW living with HIV in this setting reported relatively high internalized stigma related to HIV. While levels of experienced stigma were lower than internalized stigma, more than half of the sample had suffered some form of discrimination related to their HIV status at some point. A multi-level intervention is needed to directly address stigma and discrimination, and future research should examine the nuanced and potentially distinct roles that internalized versus experienced stigma may play on HIV-related outcomes. Such work should contemplate the multiple inequalities faced by FSW living with HIV, including not only their HIV status, but also their profession, gender, and poverty level [15], [50], [51].

Our study had several limitations. Due to the fact that we did not randomly sample women from the universe of all FSW living with HIV in Santo Domingo, our results may not be generalizable. Additionally, our cross-sectional design does not allow us to determine causal relationships between independent variables and outcomes. Our approach to recruitment via peer navigators, HIV clinics, and snowball referrals limited our ability to reach women who had not disclosed their status to anyone and were not enrolled in care; this population should be a priority for future efforts. Despite these limitations, we believe our paper makes an important contribution to the very small literature on the treatment and prevention experiences of FSW living with HIV.

Together, these baseline findings indicate the importance of the *Abriendo Puertas* intervention to address ongoing gaps in the continuity of care for both the treatment of HIV and other STI among FSW living with HIV, a key population whose health has historically been underserved. Our integrated model aims to provide multiple levels of support, advocacy, and accompaniment by working with peer navigators, clinical providers, and the FSW community to promote optimal HIV care and treatment outcomes and overall wellbeing, and to reduce ongoing transmission.
