# Correction: *Abriendo Puertas*: Baseline Findings from an Integrated Intervention to Promote Prevention, Treatment and Care among FSW Living with HIV in the Dominican Republic

**DOI:** 10.1371/journal.pone.0092480

**Published:** 2014-03-24

**Authors:** The PLOS ONE Staff

In the Funding statement, a funding organization and grant are incorrectly omitted. The Funding statement should read: “The study was funded by the U.S. Agency for International Development (http://www.usaid.gov/) under Contract No. GHH-I-00-07-00032-00, beginning September 30, 2008, and supported by the President’s Emergency Plan for AIDS Relief. We are also grateful to the Carolina Population Center (R24 HD050924) for general support (http://www.cpc.unc.edu/). The funders had no role in study design, data collection and analysis, decision to publish, or preparation of the manuscript.”

In addition, in the Acknowledgments, multiple organizations involved with the study for the article are incorrectly omitted. The Acknowledgments should read: “We thank the study participants for their time and dedication. We are also grateful to the study staff, including the leadership and team members of the Instituto Dermatalógico y Cirugia de Piel, the Movimiento de Mujeres Unidas, and the Centro de Orientacion e Investigacion Integral.”
